# Supplementary material for: Gene co-expression network connectivity is an important determinant of selective constraint
Source: PLoS Genet. 2017 Apr 13;13(4):e1006402. doi: 10.1371/journal.pgen.1006402 (PMC5407845; doi:10.1371/journal.pgen.1006402)
Supplement: S1 Table — θπ, Tajima’s D, θ0-fold/θ4-fold and dN/dS in groups corresponding to Fig 5, and the summary of Wilcoxon rank sum test (location shift and 95% confidence interval) between two groups of estimates shown in Fig 5. (DOCX) [file pgen.1006402.s003.docx]

**S1 Table.** Statistic summary (median and central 95% range) for four measures of sequence evolution: θ_π_, Tajima’s D, θ_0-fold_/θ_4-fold_ and dN/dS in groups corresponding to Figure 5, and the summary of Wilcoxon rank sum test (location shift and 95% confidence interval) between two groups of estimates shown in Figure 5.

| **Statistics of sequence diversity and divergence** | | |  |  |  |
| --- | --- | --- | --- | --- | --- |
|  | n | θ_π_ | Tajima’s D | θ_0-fold_/θ_4-fold_ | d_N_/d_S_ |
| non-eGene | 16065 | 0.0087  (0.0026-0.0287) | -1.5122  (-2.5508- -0.3141) | 0.3402  (0.0593-4.3145) | 0.2951  (0.0010-1.5137) |
| eGene-distant | 1050 | 0.0097  (0.0029-0.0469) | -1.4323  (2.5392 - -0.0529) | 0.4188  (0.0614-4.7226) | 0.3264  (0.0010-2.2527) |
| eGene-local | 5191 | 0.0104  (0.0036-0.0288) | -1.2350(-2.3275- -0.0586) | 0.3714  (0.0602-4.9811) | 0.3055  (0.0010-1.7916) |
|  | n | θ_π_ | Tajima’s D | θ_0-fold_/θ_4-fold_ | d_N_/d_S_ |
| eGene:core | 138 | 0.0091  (0.0025-0.0324) | -1.5032  (-2.5404- -0.1691) | 0.2910  (0.0451-6.1055) | 0.2631  (0.0010-1.3240) |
| eGene:non-core | 6103 | 0.0104  (0.0034-0.0324) | -1.2625  (-2.3658- -0.0554) | 0.3794  (0.0614-4.9222) | 0.3091  (0.0010-1.8446) |
| non-eGene:core | 1657 | 0.0078  (0.0022-0.0214) | -1.6473  (-2.6649- -0.4013) | 0.2899  (0.0492-3.2742) | 0.2549  (0.0010-1.2893) |
| non-eGene:non-core | 14408 | 0.0088  (0.0027-0.0299) | -1.4944  (-2.5329- -0.3009) | 0.3472  (0.0609-4.4685) | 0.3009  (0.0010-1.5353) |
| **Wilcoxon rank sum test** | |  |  |  |  |
|  |  | θ_π_ | Tajima’s D | θ_0-fold_/θ_4-fold_ | d_N_/d_S_ |
| non-eGene vs. eGene-distant |  | -0.0011  (-0.0015- -0.0008)*** | -0.0784  (-0.1202- -0.0366)*** | -0.0558  (-0.0776- -0.0346)*** | -0.0286  (-0.0448- -0.0128)*** |
| non-eGene vs. eGene-local |  | -0.0017  (-0.0019- -0.0016)*** | -0.2635  (-0.2823- -0.2446)*** | -0.0302  (-0.0392- -0.0213)*** | -0.0114  (-0.0190- -0.0038)** |
| eGene-distant vs. eGene-local |  | -0.0005  (-0.0009- -0.0001)** | -0.1849  (-0.2283- -0.1413)*** | 0.0265  (0.0032-0.0501)* | 0.0166  (-4.5e-05- 3.4e-02)n.s. |
|  |  | θ_π_ | Tajima’s D | θ_0-fold_/θ_4-fold_ | d_N_/d_S_ |
| Non-eGene: core vs. non-core |  | -0.0008  (-0.0011- -0.0007)*** | -0.1421  (-0.1732- -0.1108)*** | -0.0488  (-0.0618- -0.0360)*** | -0.0620  (-0.1028- -0.0212)** |
| eGene: core vs. non-core |  | -0.0011  (-0.0020- -0.0003)* | -0.1708  (-0.2875- -0.0546)** | -0.0739  (-0.1237- -0.0256)** | -0.0432  (-0.0544- -0.0321)*** |

n: number of genes

ns: non significant (P>0.05)

*P<0.05

**P<0.01

***P<0.001
