# Supplementary figures and images for: Gene co-expression network connectivity is an important determinant of selective constraint
Source: PLoS Genet. 2017 Apr 13;13(4):e1006402. doi: 10.1371/journal.pgen.1006402 (PMC5407845; doi:10.1371/journal.pgen.1006402)

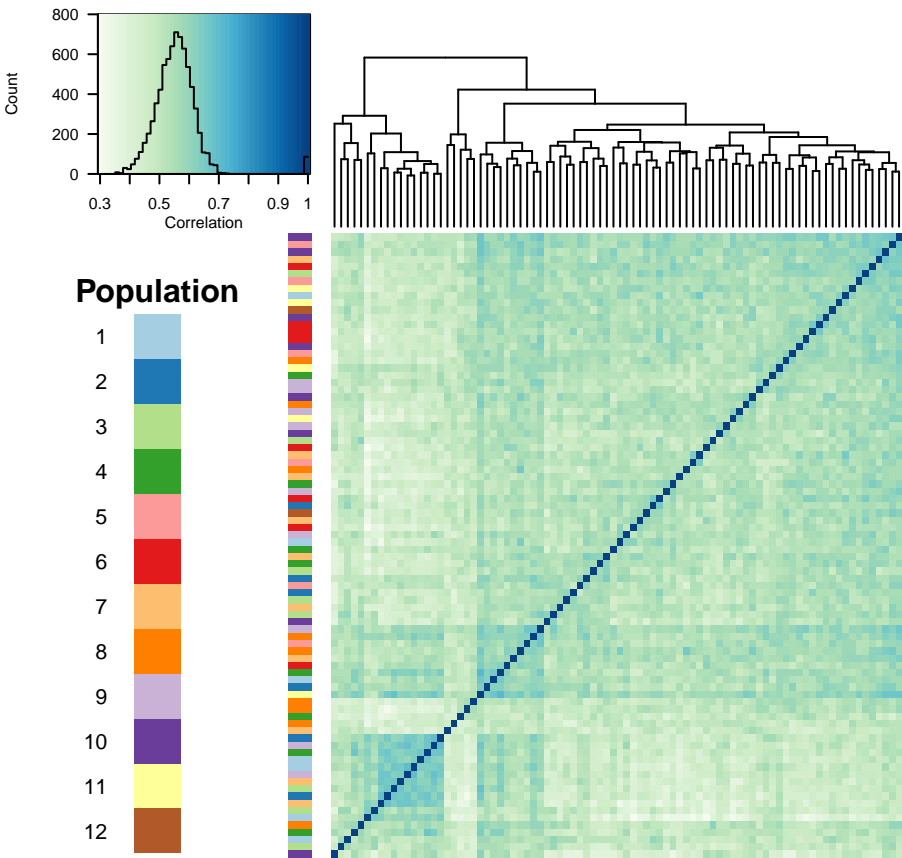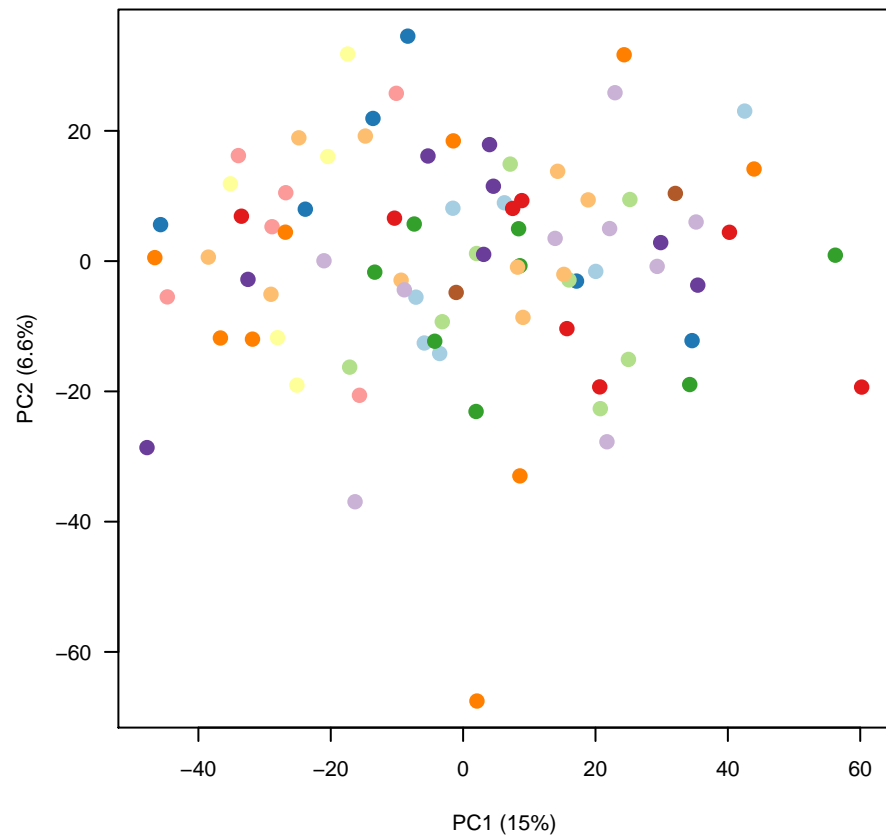

Supplement: S1 Fig — (Left) Heatmap of the sample correlation matrix based on the 500 most variably expressed genes. Darker colour indicates higher correlation. The coloured bar represents the populations the genotypes belong to. (Right) The two first principal components from a principal component analysis (PCA) based on all genes. Again, colours represent the genotype population. The percentages in the axis labels indicate the amount of variance explained by each component. (PDF) [file pgen.1006402.s004.pdf]

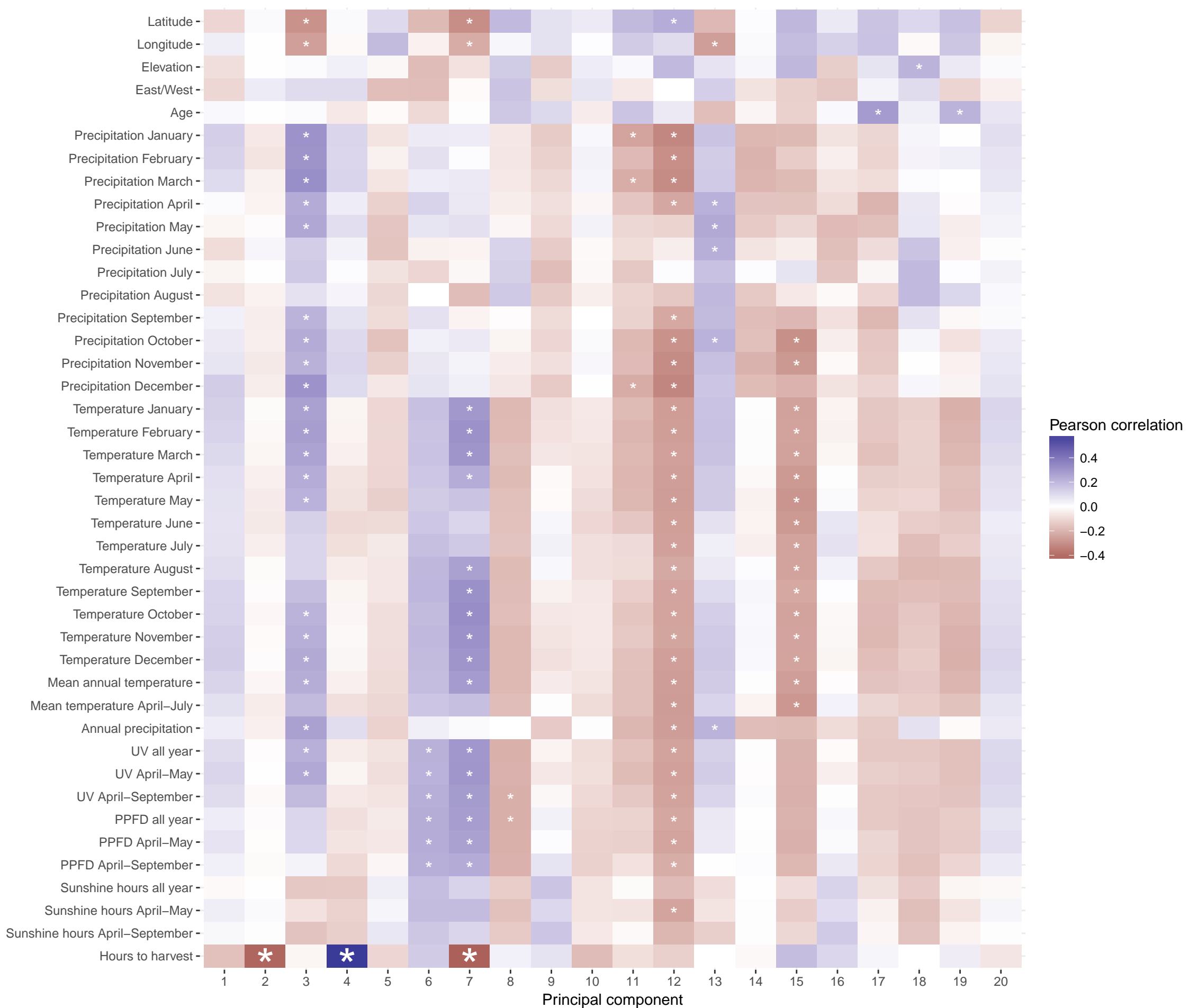

Supplement: S2 Fig — The values in each tile represent the Pearson correlation between the gene expression PC (x-axis) and environmental variable (y-axis). Small asterisks represent a nominal p-value < 0.05 while large asterisks represent Benjamini-Hochberg (BH) adjusted p-values < 0.05. The only factor with significant correlations to expression PCs was “Hours to harvest”, which is the number of hours into the sampling period that the buds were harvested. It was significantly associated with PC4 (BH-adjusted p = 4.6×10^−6^), PC7 (BH-adjusted p = 0.030) and PC2 (BH-adjusted p = 0.033). (PDF) [file pgen.1006402.s005.pdf]

**SwAsp**

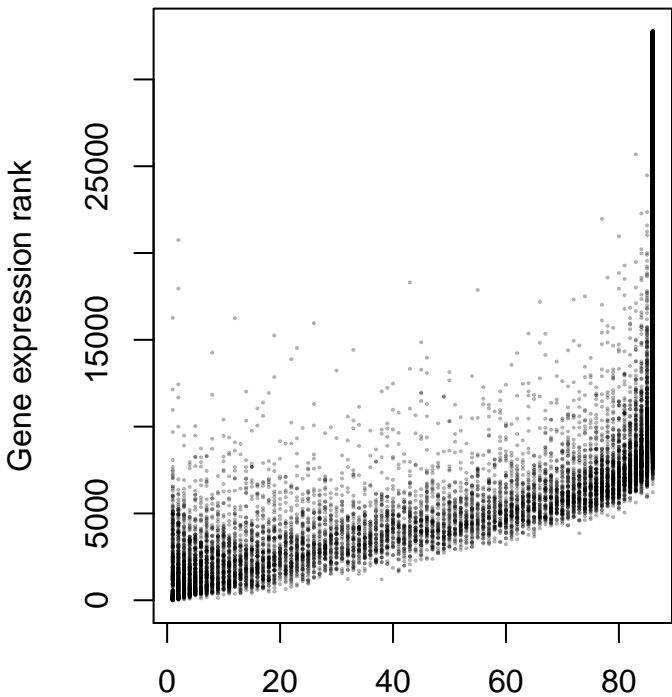

**UmAsp**

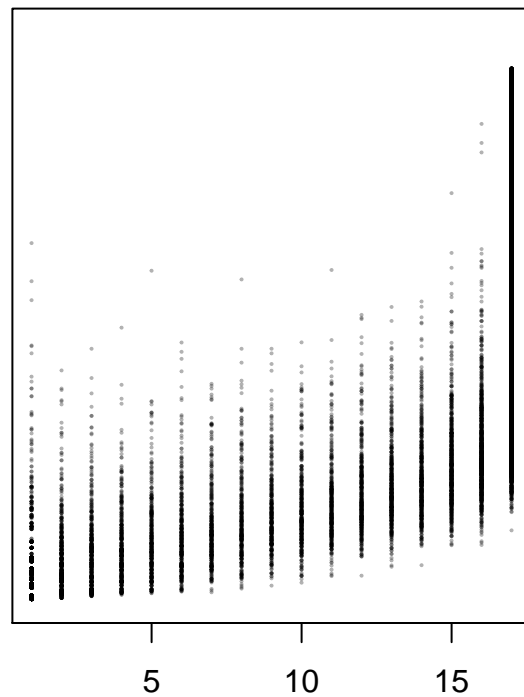

Number of genotypes

Supplement: S3 Fig — The number of genotypes that a gene was expressed in was determined by counting the number of genotypes with non-zero expression for each gene. The gene expression ranks were calculated by ranking the mean gene expression values where the mean was calculated only considering samples with non-zero expression. (PDF) [file pgen.1006402.s006.pdf]

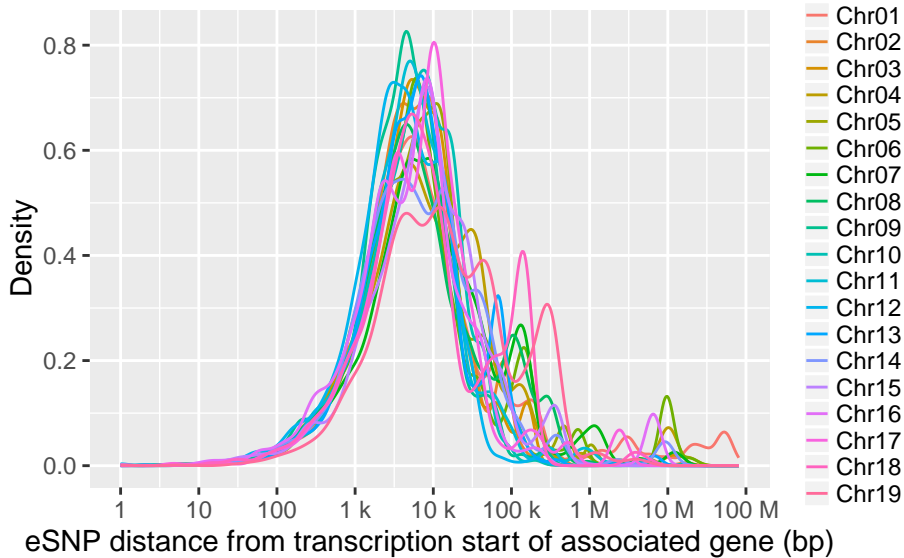

Supplement: S4 Fig — (PDF) [file pgen.1006402.s007.pdf]

Number of eQTLs (5% BH FDR)

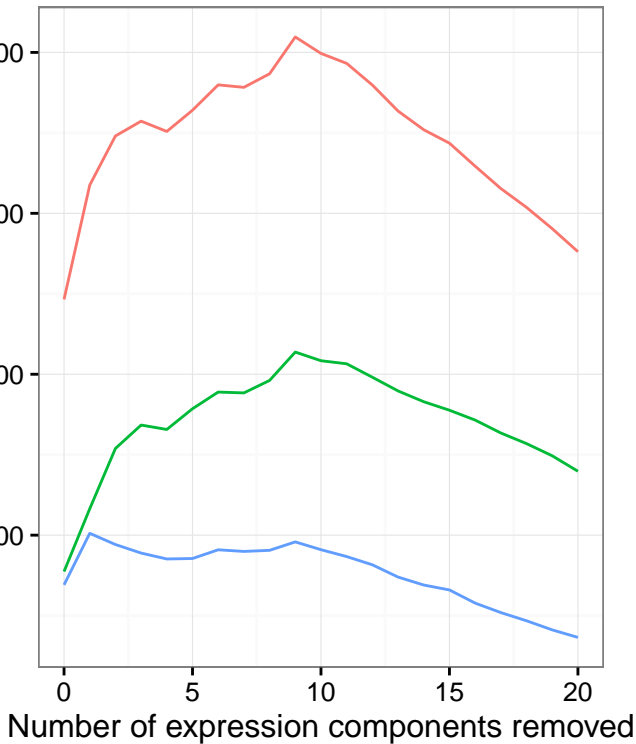

eQTL type

- All
- Local
- Distant

Supplement: S5 Fig — The y-axis shows the number of eQTLs detected at 5% FDR (prior to the empirical FDR calculation). The x-axis shows the number of principal components regressed out of the gene expression data. Distant eQTLs are defined as eQTLs where the gene and the SNP are located on different scaffolds. (PDF) [file pgen.1006402.s008.pdf]

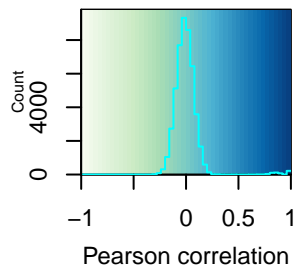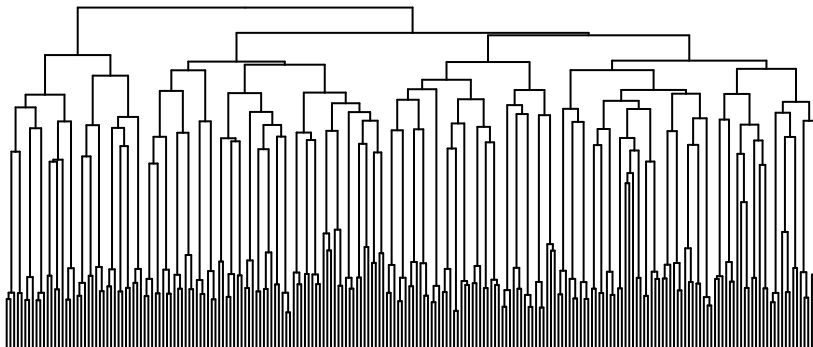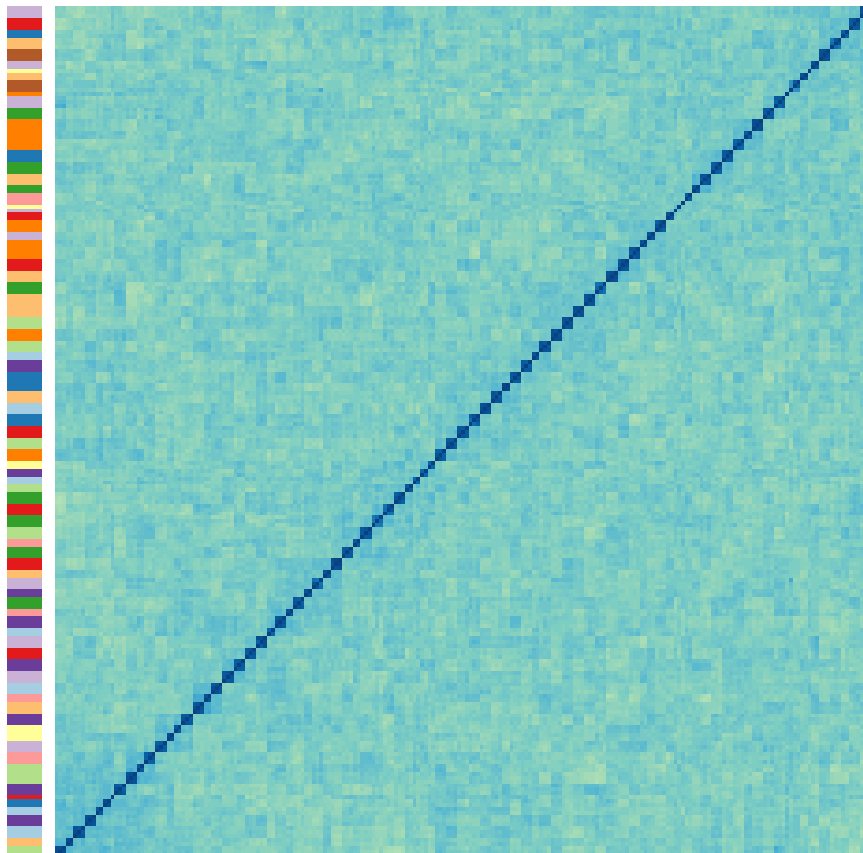

Supplement: S6 Fig — The 500 most variable genes in the original expression data were used for calculating the sample correlations (i.e. the same genes as in Fig 1D). The colour bar represents the population of the samples with the same colour scheme as in Fig 1D. The small clusters on the diagonal represent genotype replicates. (PDF) [file pgen.1006402.s009.pdf]

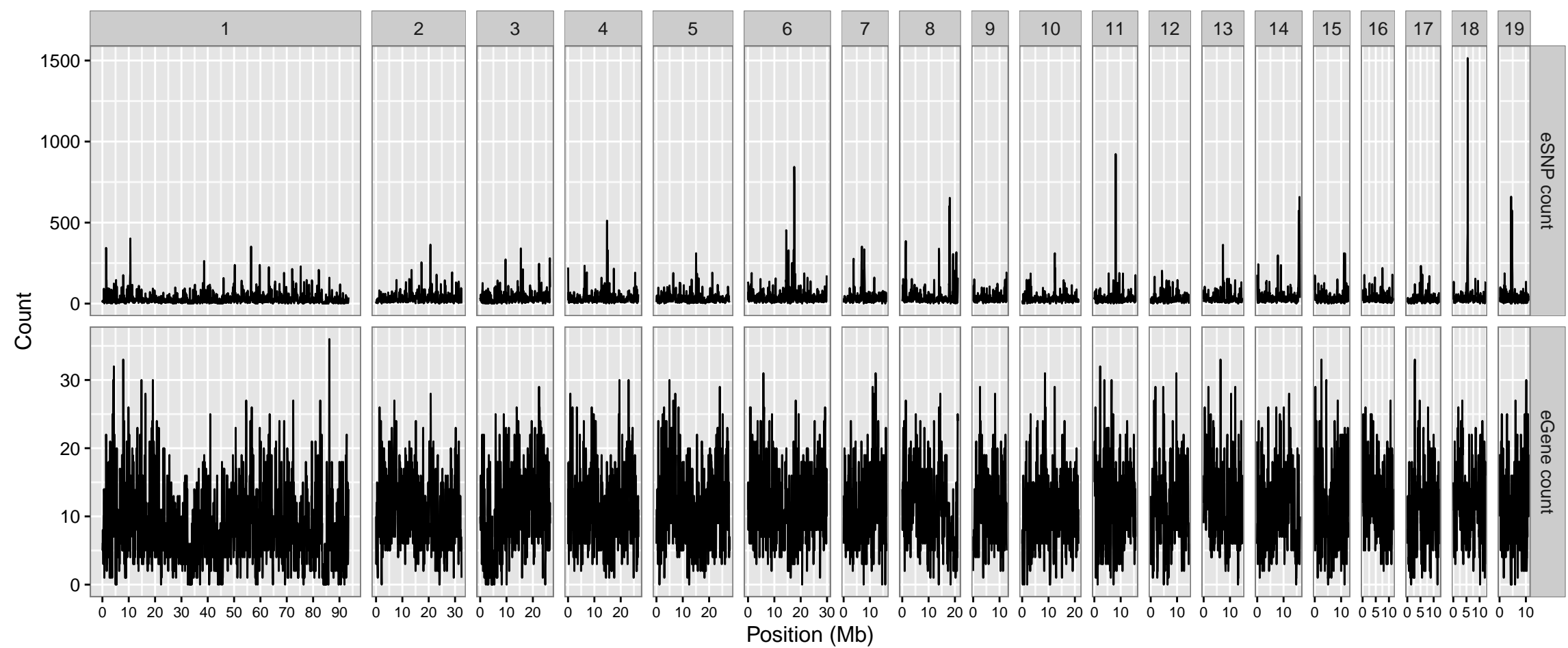

Supplement: S7 Fig — The upper panel shows the number of eSNPs in genomic windows of 100 kb for each of the 19 chromosomes. The lower panel shows the number of unique genes that are associated to each genomic window (nominal eQTL p-value < 1×10^−6^). (PDF) [file pgen.1006402.s010.pdf]

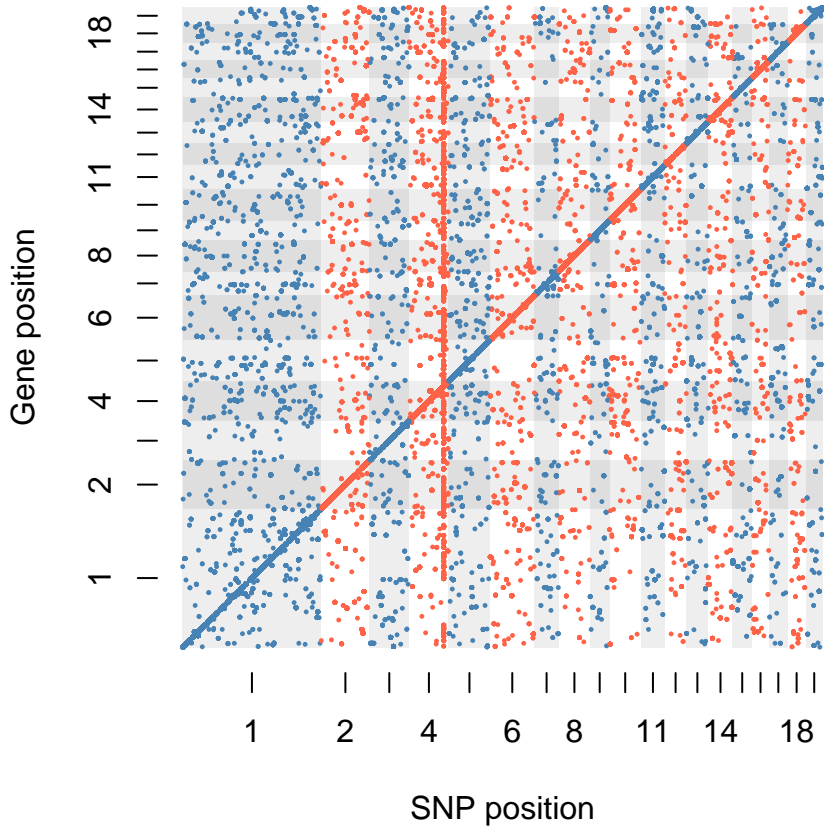

Supplement: S8 Fig — A clear hotspot can be seen at the end of chromosome 4. (PDF) [file pgen.1006402.s011.pdf]

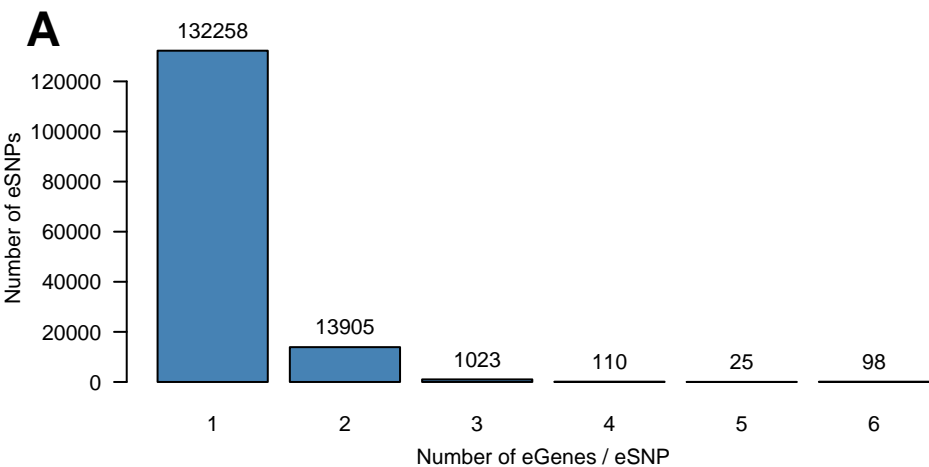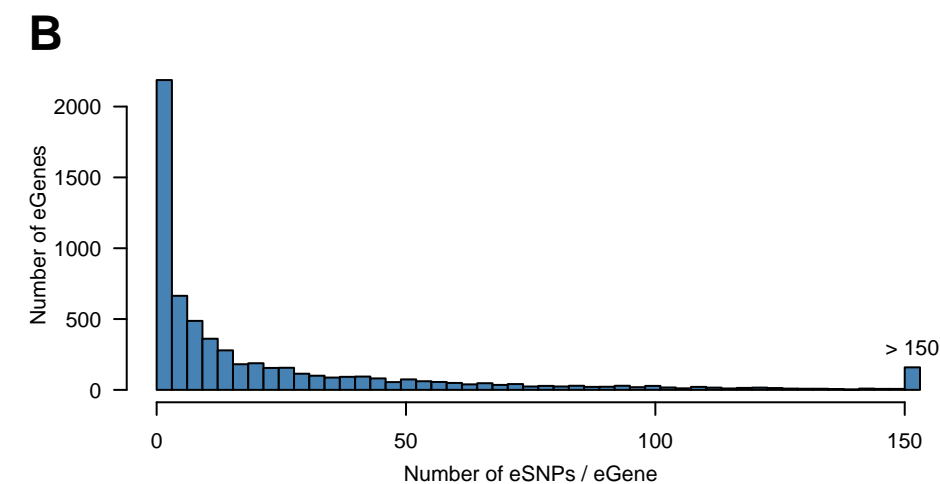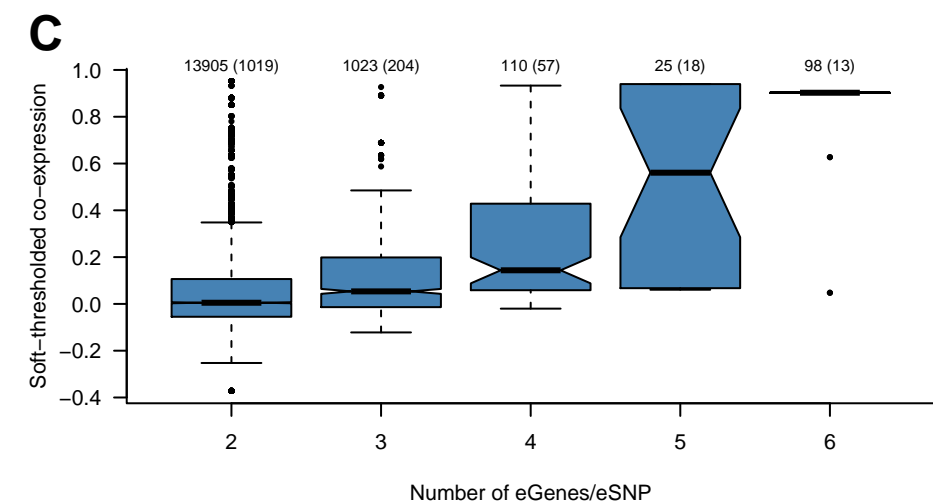

Supplement: S9 Fig — (A) Bar plot of the number of associated eGenes per eSNP. (B) Histogram of the number of associated eSNPs per eGene. (C) The mean co-expression for genes associated with the same eSNP. The numbers on the x-axis represents the number of eGenes that a single eSNP is associated to, and the numbers above the boxes shows the total number of eSNPs that are represented, and in parentheses are the number of unique genes that they are associated with. (PDF) [file pgen.1006402.s012.pdf]

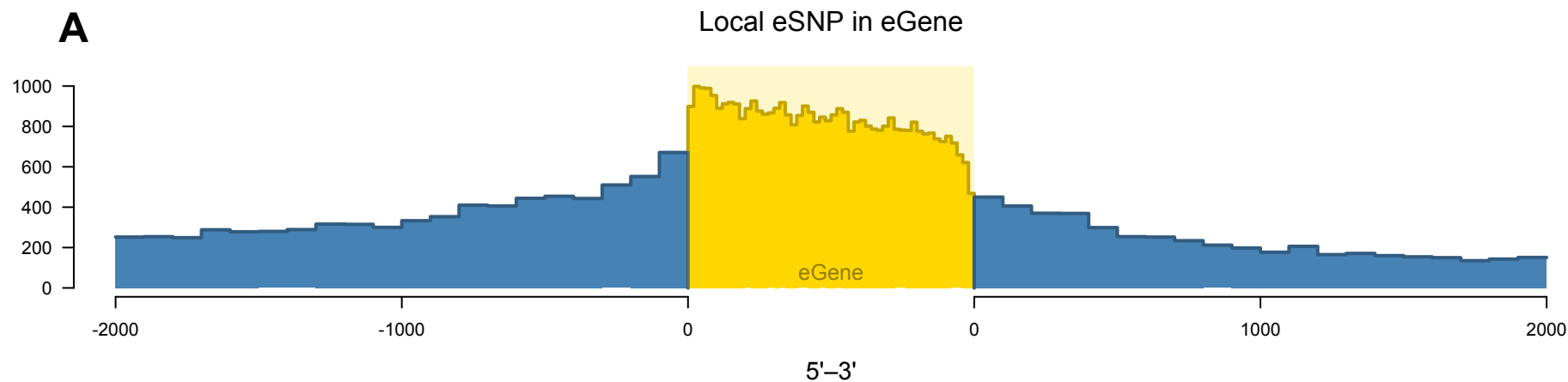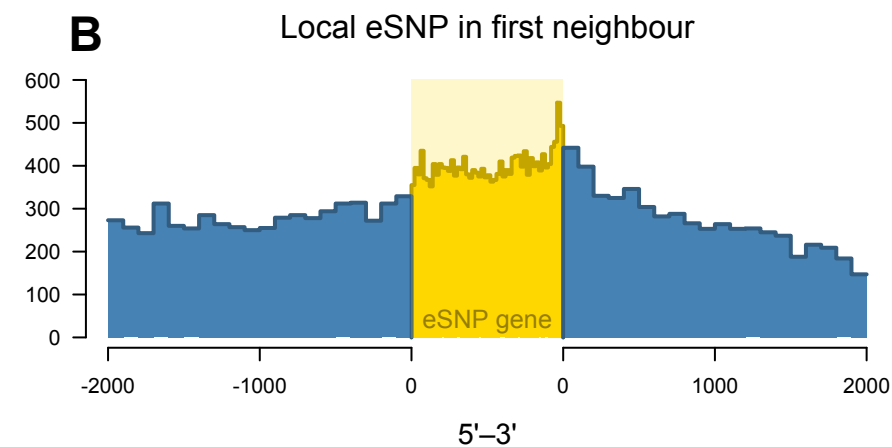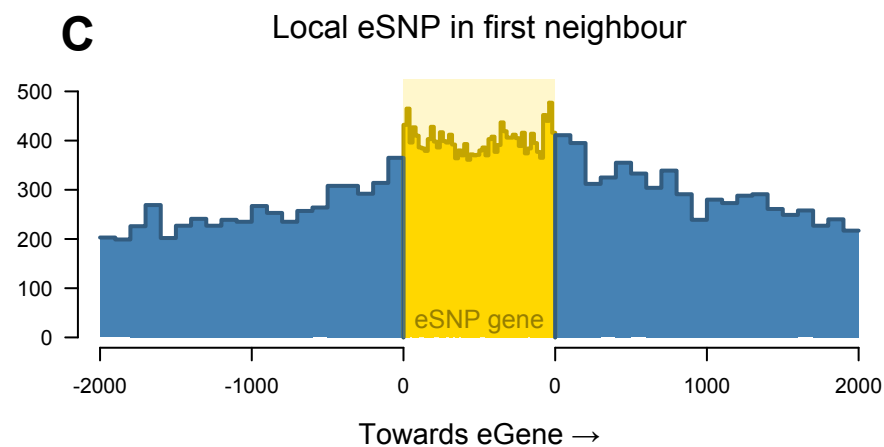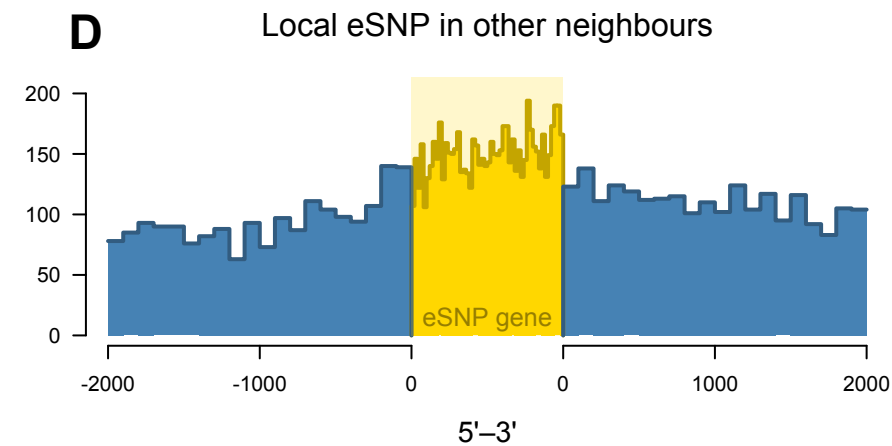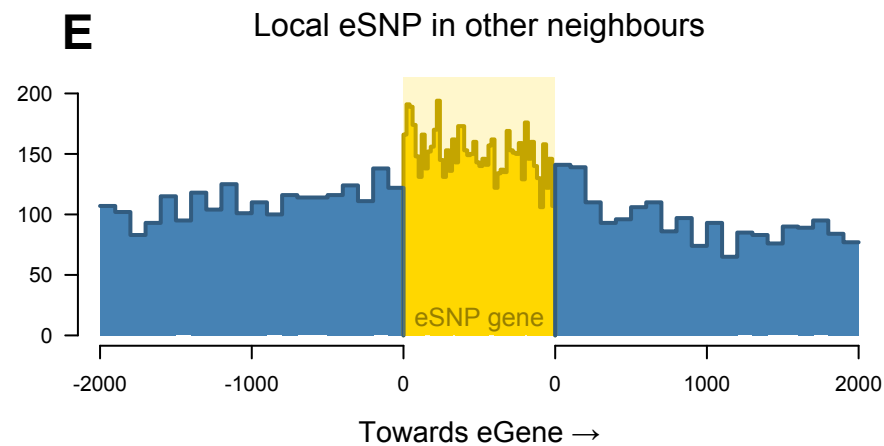

Supplement: S10 Fig — Local eSNPs were counted in cases where the eSNP was located in the gene it was associated with (A) as well as when it was located in a direct neighbour of the eGene (B, C) or in a gene further away from the eGene (D, E). For cases when the eSNP was located in neighbouring genes (B-E), this was plotted both in the direction of the gene (B, D) and relative to the eGene (C, E). Up and downstream regions were divided into 1 kbp bins while the intergenic region was normalised to 20 bins. In other words, up and downstream counts are not directly comparable to intragenic counts. (PDF) [file pgen.1006402.s013.pdf]

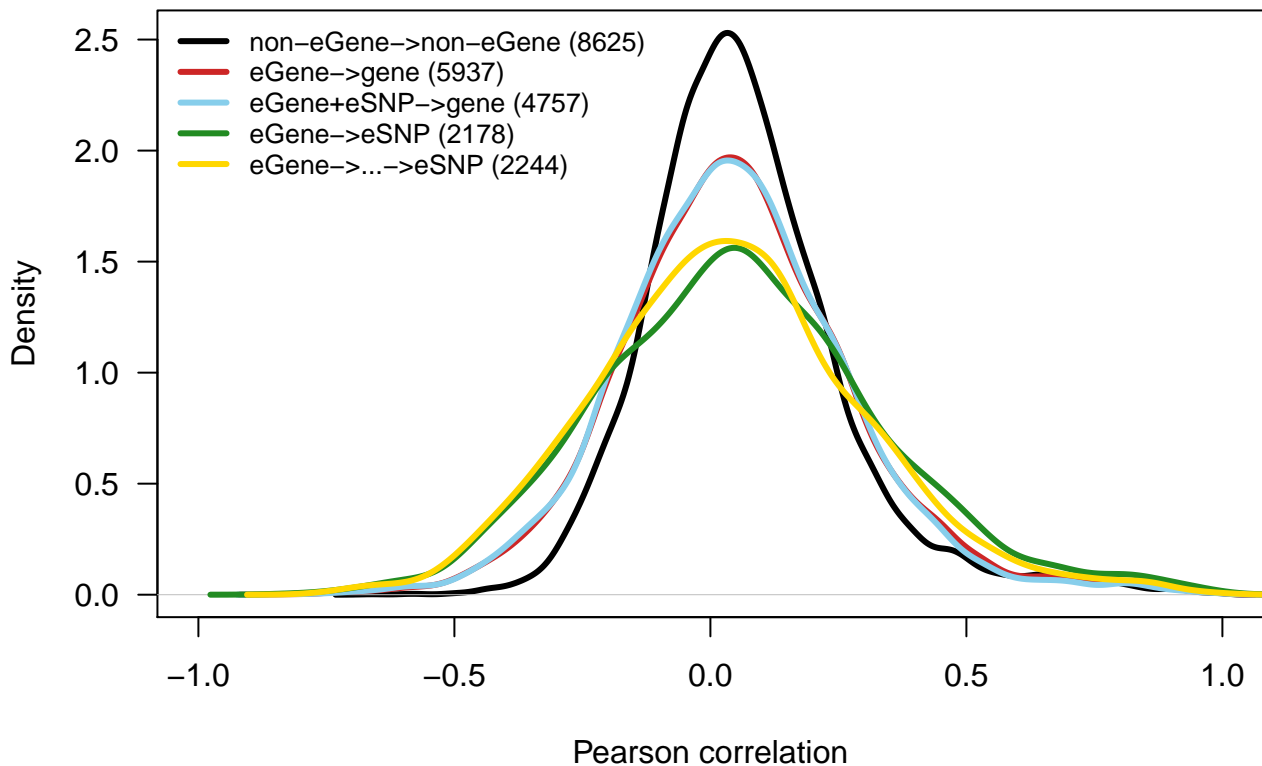

Supplement: S11 Fig — Neighbouring genes in the genome were divided into five different categories: non-eGene—non-eGene, eGene—gene, eGene with eSNP proximal to it—gene, eGene—first neighbour harbouring the eSNP, and eGene—neighbour further away harbouring the eSNP. The numbers in parenthesis in the legend represent the number of pairs in each category. (PDF) [file pgen.1006402.s014.pdf]

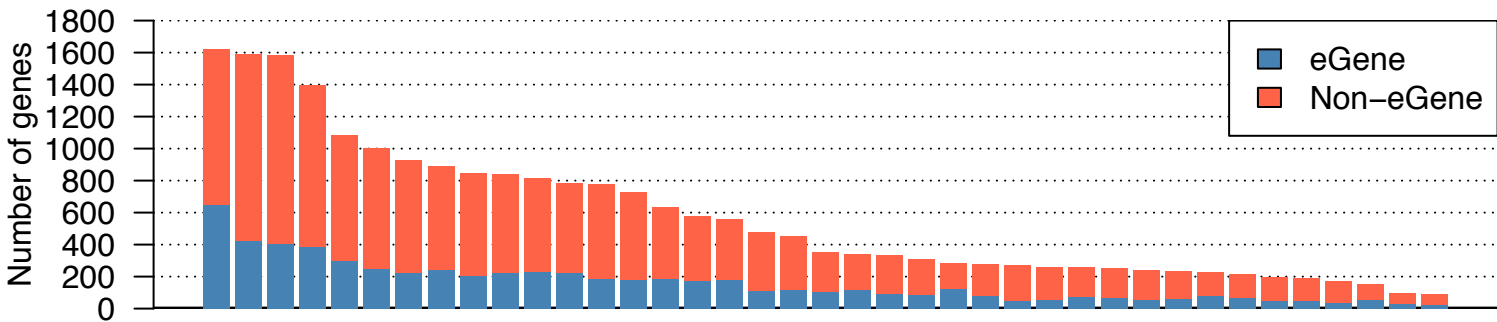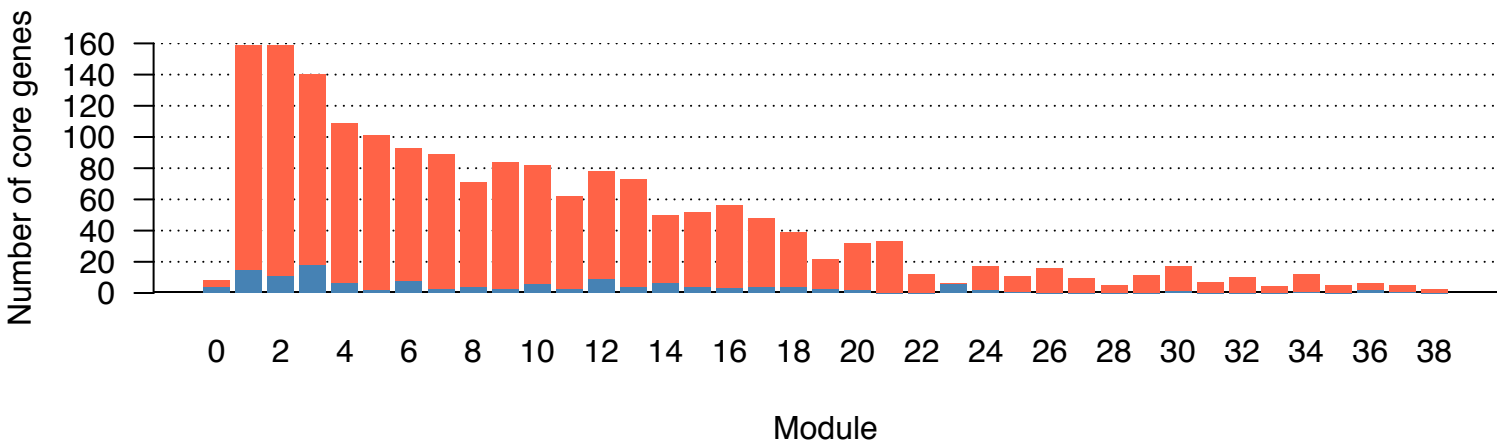

Supplement: S12 Fig — Each bar is divided into eGene and non-eGene assignments. Module 0 contains genes not assigned to any module. (PDF) [file pgen.1006402.s015.pdf]

Expression variance

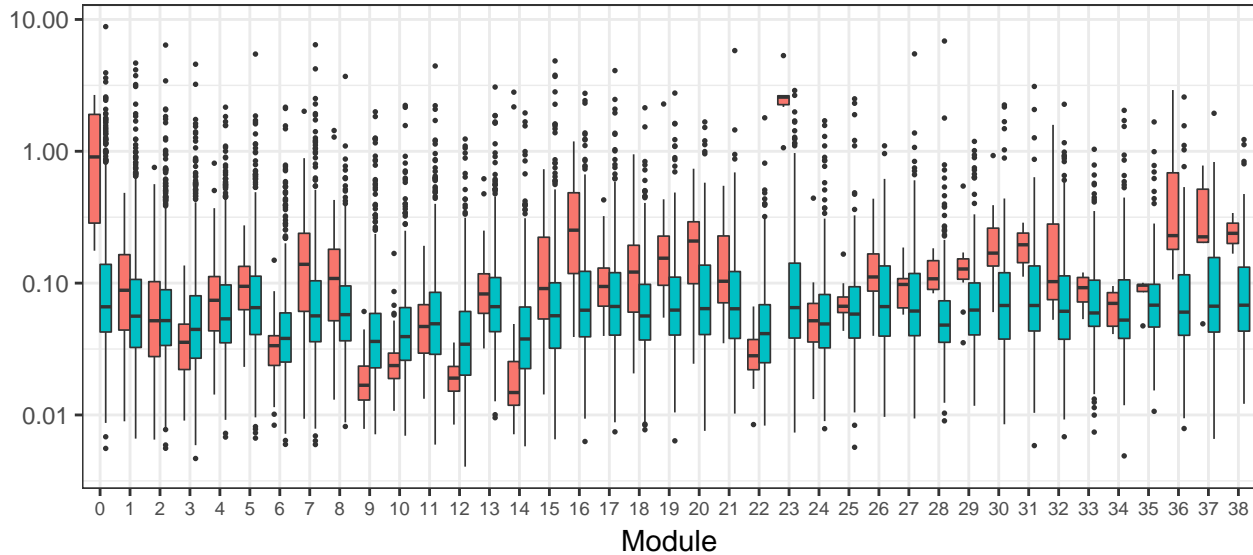

Core  
Non-core

Supplement: S13 Fig — (PDF) [file pgen.1006402.s016.pdf]

Co-expression connectivity

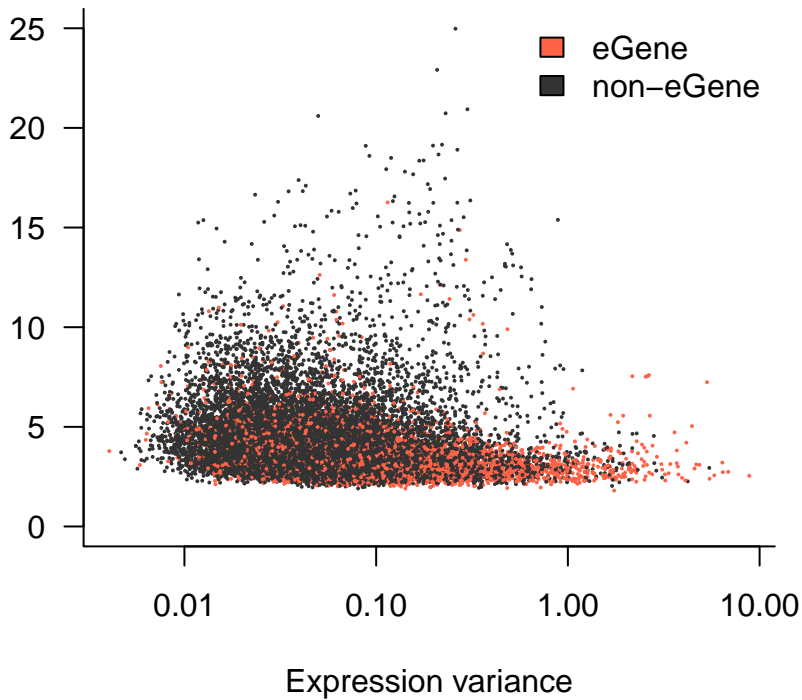

Supplement: S14 Fig — eGenes are indicated by red points. (PDF) [file pgen.1006402.s017.pdf]

Co-expression connectivity

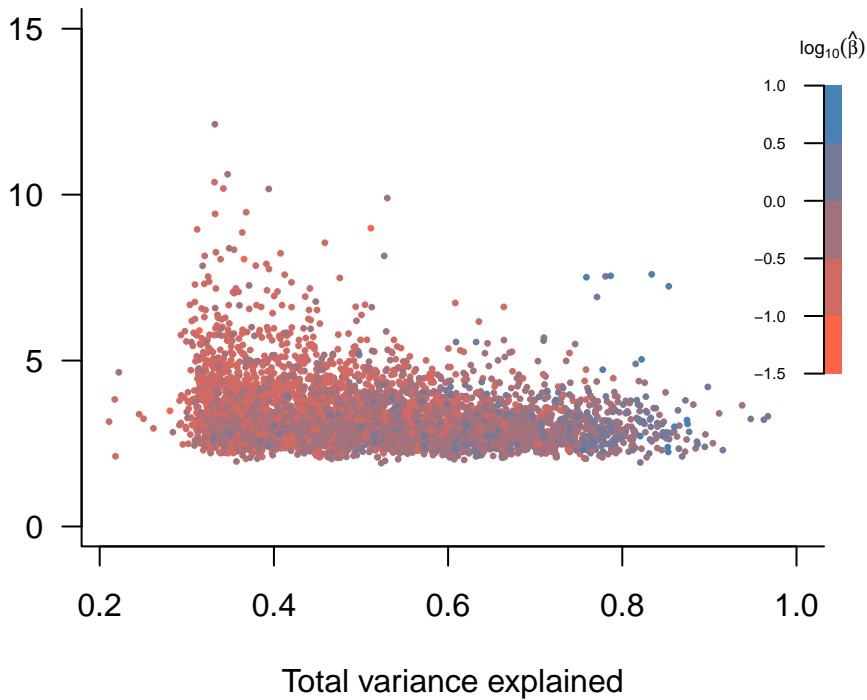

Supplement: S15 Fig — The variance explained is the adjusted variance explained based on all eQTL associated with each gene. (PDF) [file pgen.1006402.s018.pdf]

**A**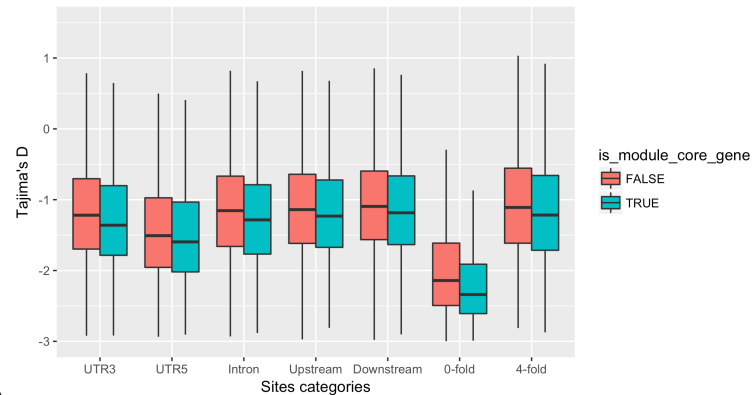**B**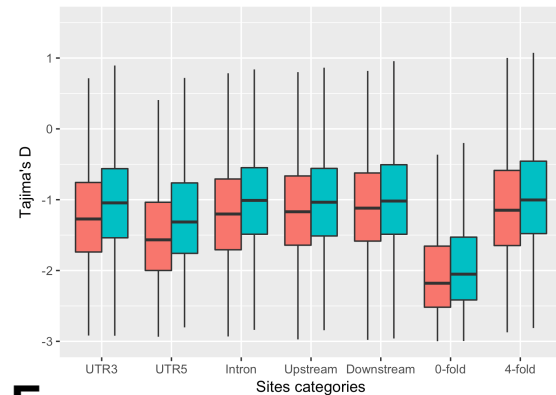**C**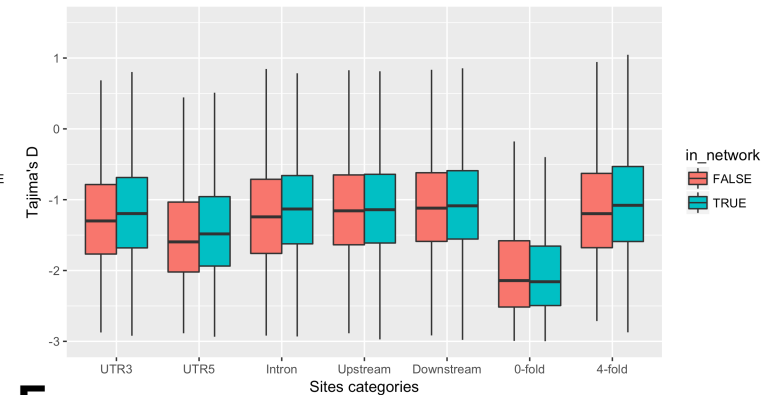**D**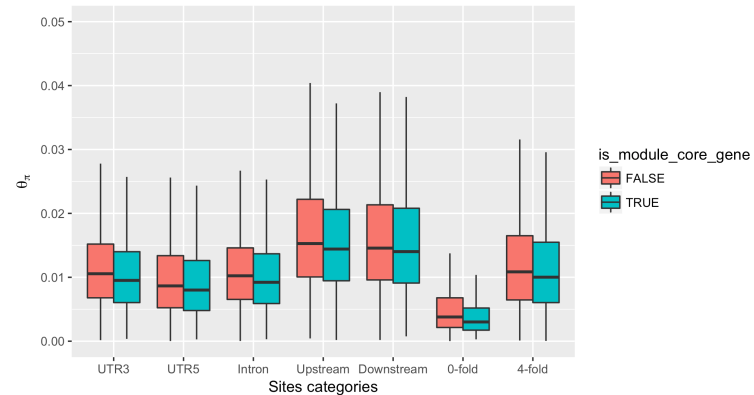**E**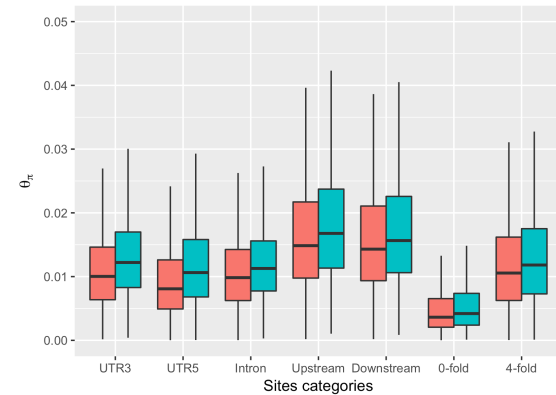**F**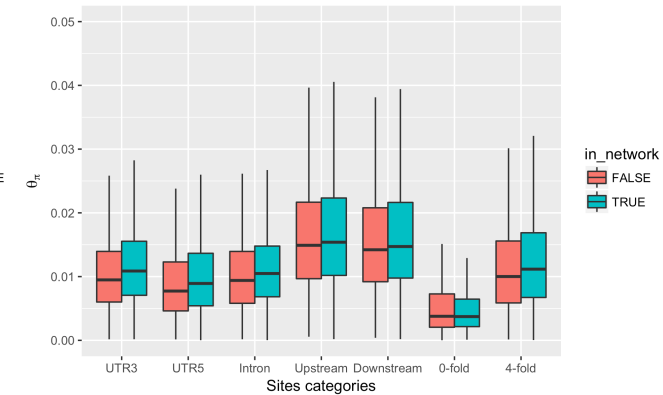

Supplement: S16 Fig — Tajima’s D was compared between (A) core/non-core genes, (B) eGenes/non-eGenes, and (C) whether the gene was included in the co-expression network or not. The same comparisons were also done for nuclotide diversity (D-F). Upstream and downstream are 1 kbp away from the gene start and end, respectively. 0-fold and 4-fold refers to 0-fold and 4-fold degenerate sites, respectively. (PDF) [file pgen.1006402.s019.pdf]

**A**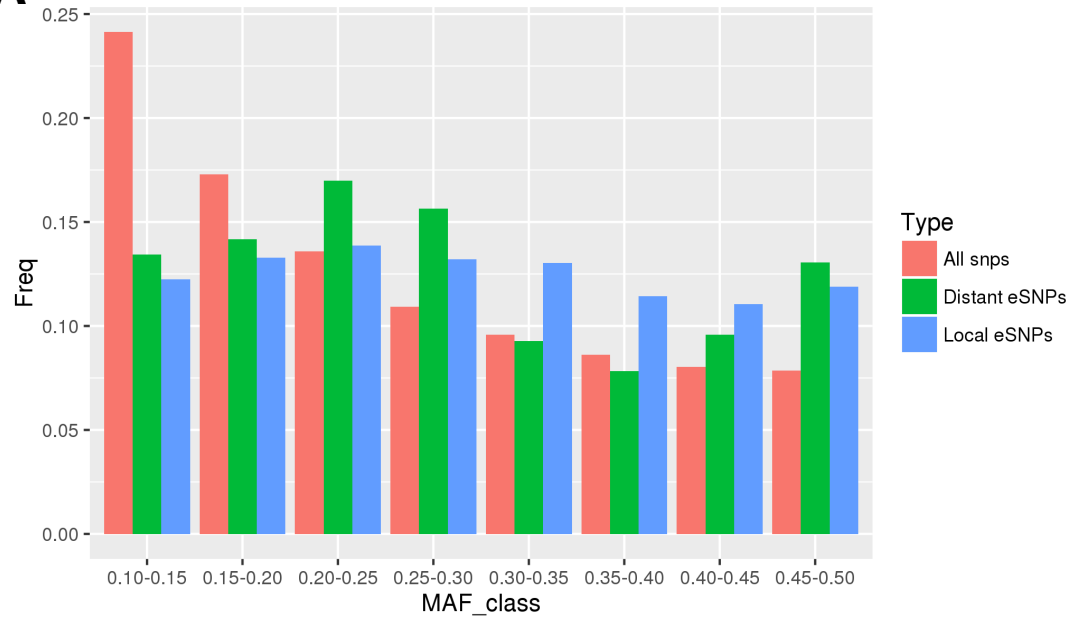**B**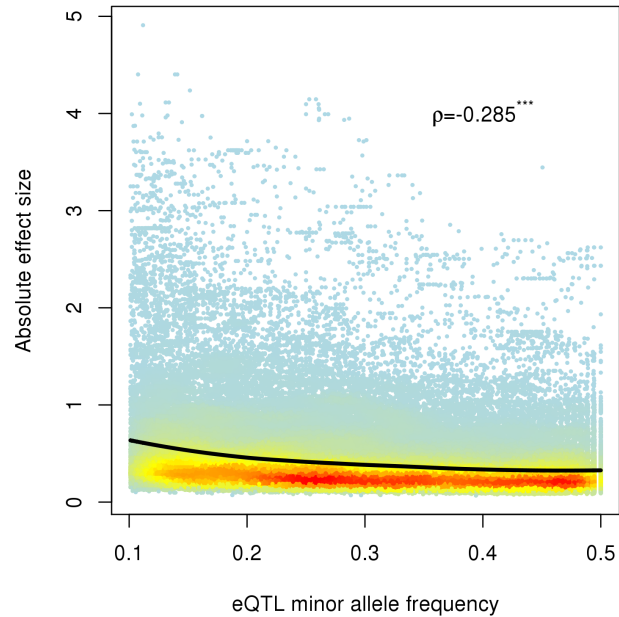

Supplement: S17 Fig — (A) Comparison of minor allele frequency between all SNPs, distant eSNPs and local eSNPs. (B) The relationship between minor allele frequency and effect size (absolute value of beta) of eQTLs. (PDF) [file pgen.1006402.s020.pdf]

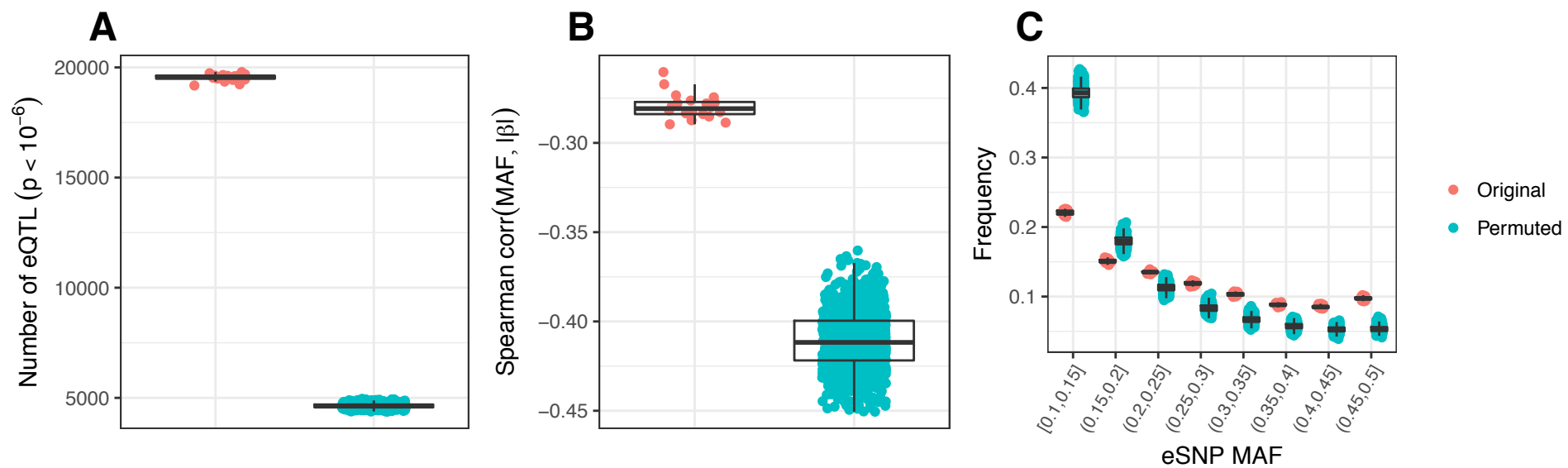

Supplement: S18 Fig — eQTL permutations were done by selecting 20 random subsets of 150,000 SNPs each. Each of these sets were associated with gene expression levels, and in addition, 50 permutations were performed for each set by shuffling the genotype labels of the SNP data, resulting in a total of 1,000 permuted eQTL sets. (A) The number of SNPs for the nominal p-value threshold 10^-6 (the same initial threshold that was used for the original associations). (B) The Spearman correlation between minor allele frequency and absolute effect size for the 20 sets and their corresponding permutations. (C) Allele frequency spectra for the 20 subsets and their permutations. (PDF) [file pgen.1006402.s021.pdf]

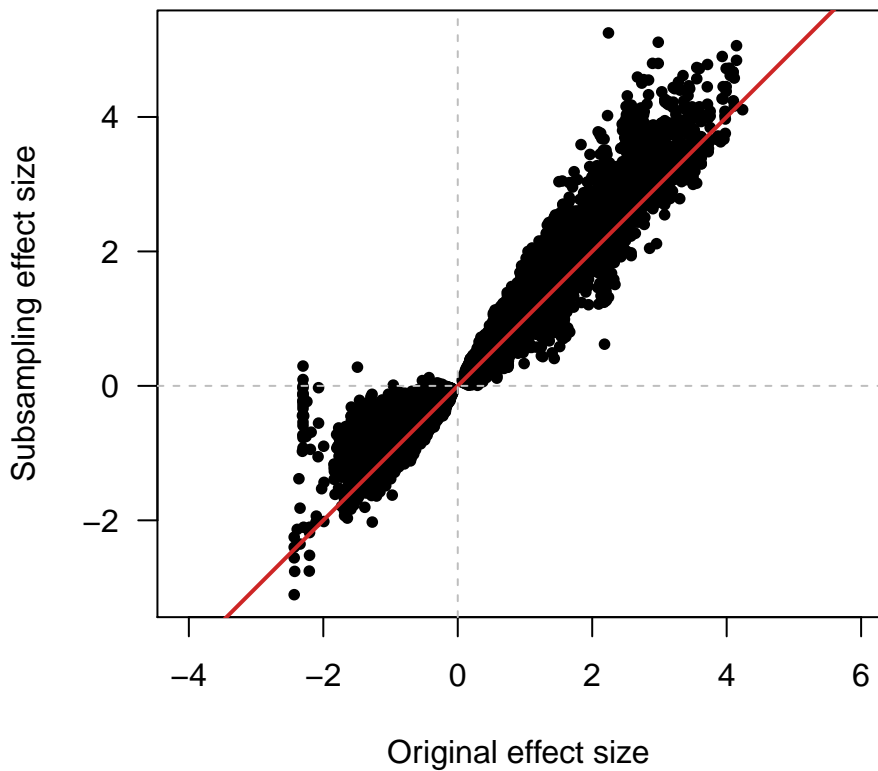

Supplement: S19 Fig — The Pearson correlation is 0.98 (df = 157,020). (PDF) [file pgen.1006402.s022.pdf]

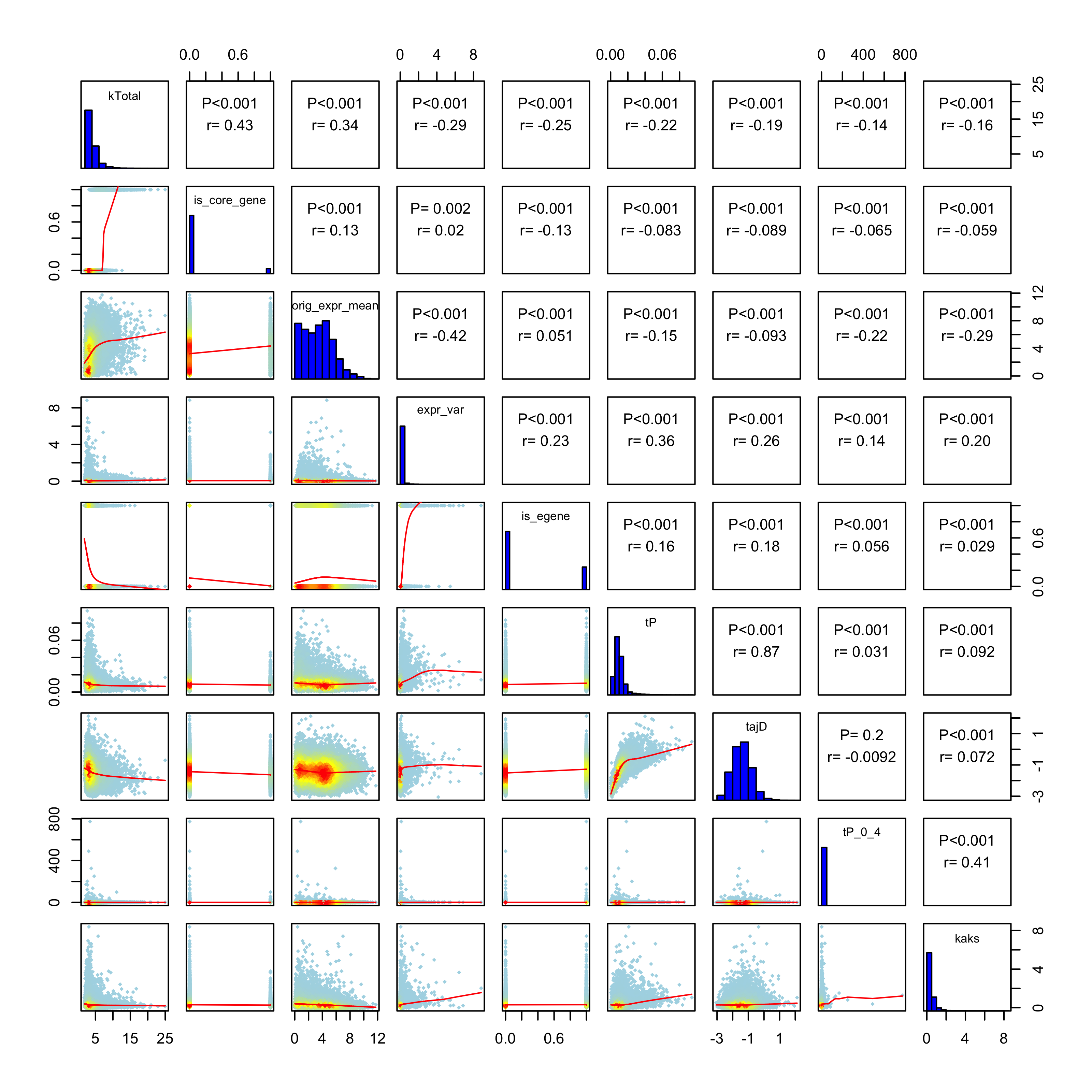

Supplement: S20 Fig — Scatter plots (lower off-diagnal, the red-to-yellow-to-blue gradient indicates decreased density of observed events at a give location in the graph) and correlations with probability values (upper off-diagonal, spearman’s rank correlations) for measures of gene expression and sequence evolution: kTotal, the total (global) connectivity in the network; is_core_gene, gene is part of module core or not; orig_expr_mean, mean expression before hidden confounder removal; orig_expr_var, expression variance before hidden confounder removal; is_egene, gene with eQTLs or not; tP, pairwise nucleotide diversity; tajD, Tajima’s D; tP_0_4, the ratio of pairwise nucleotide diversity at zero-fold non-synonymous and four-fold synonymous sites; kaks, dN/dS. (PNG) [file pgen.1006402.s023.png]

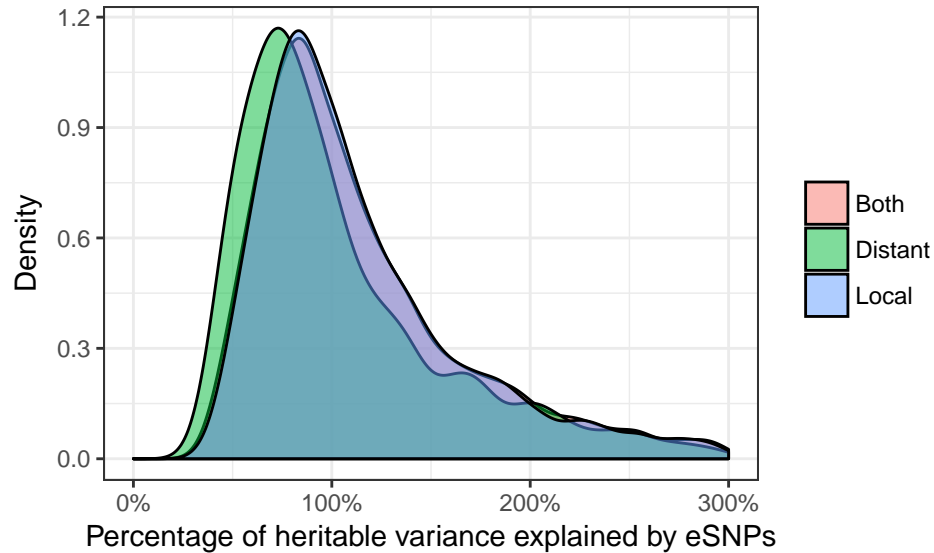

Supplement: S21 Fig — This figure assumes that eSNPs only account for heritable variation, explaining why some of the eSNPs explain more than 100% of the heritability. The x-axis has been truncated at 300%. (PDF) [file pgen.1006402.s024.pdf]

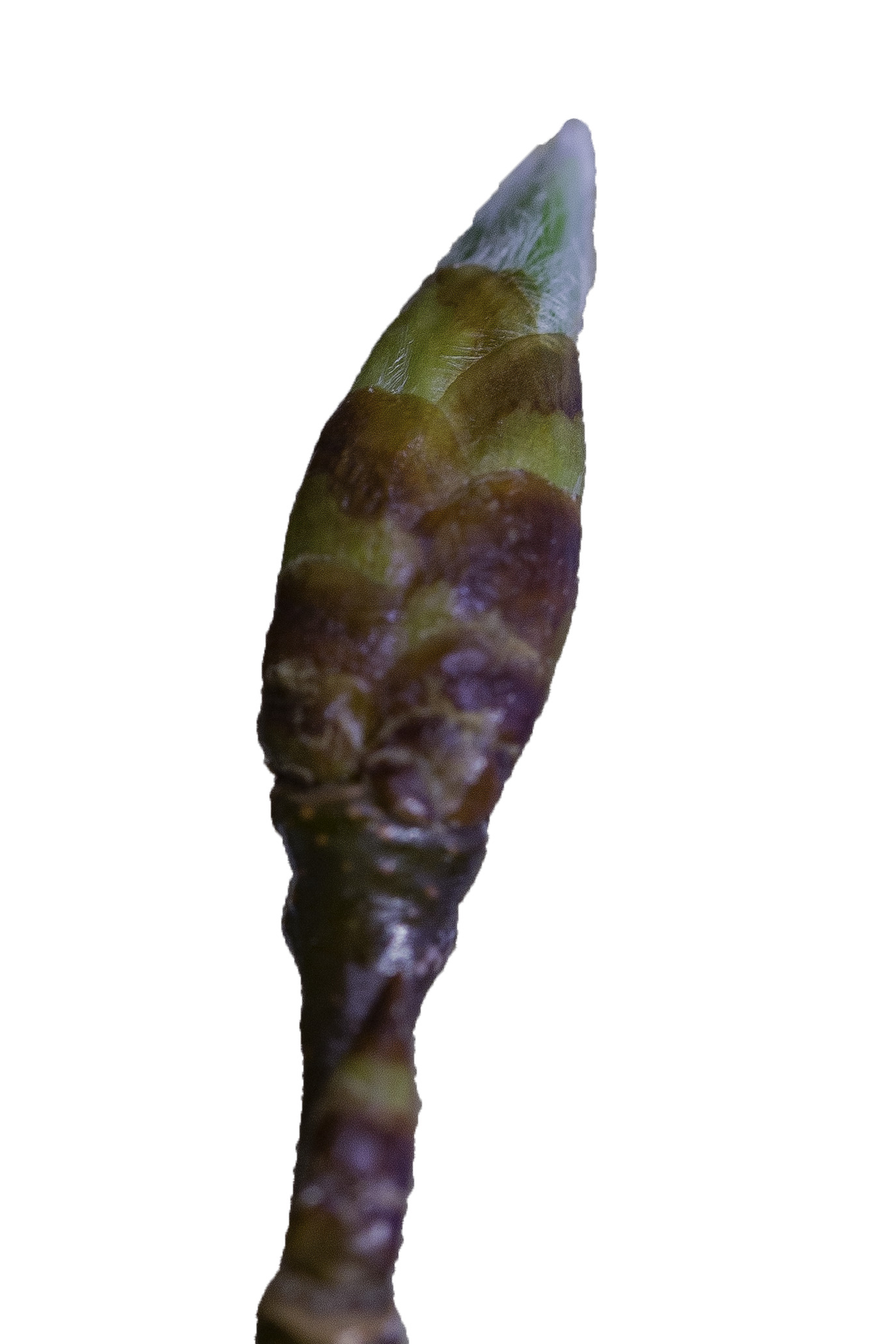

Supplement: S22 Fig — (JPG) [file pgen.1006402.s025.jpg]

**A**

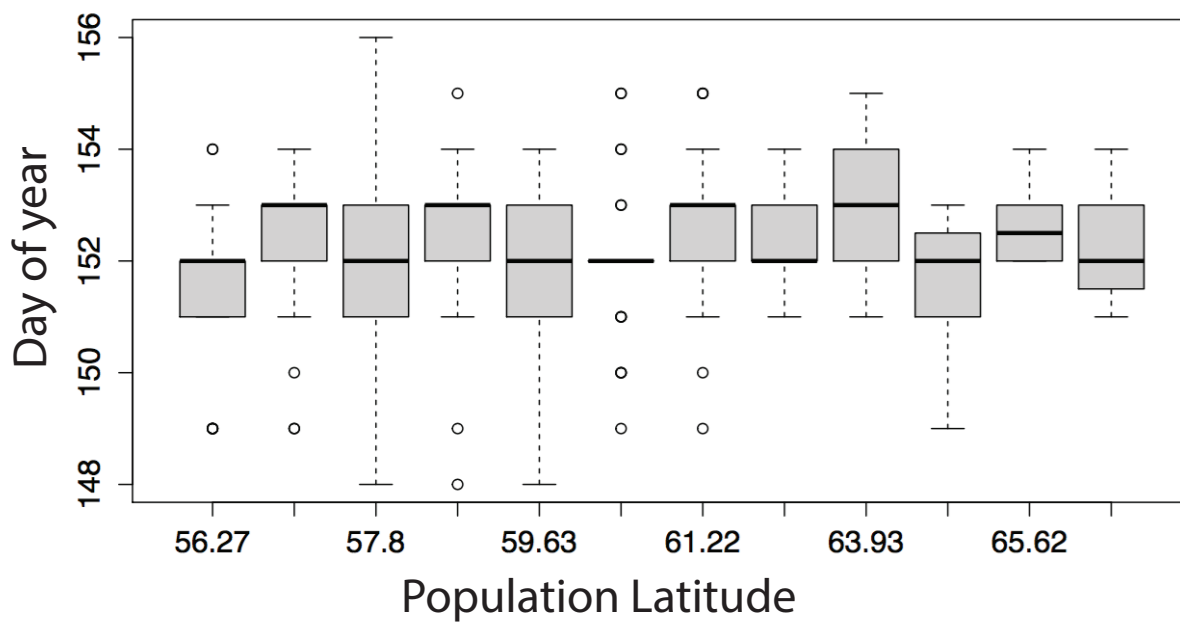

**B**

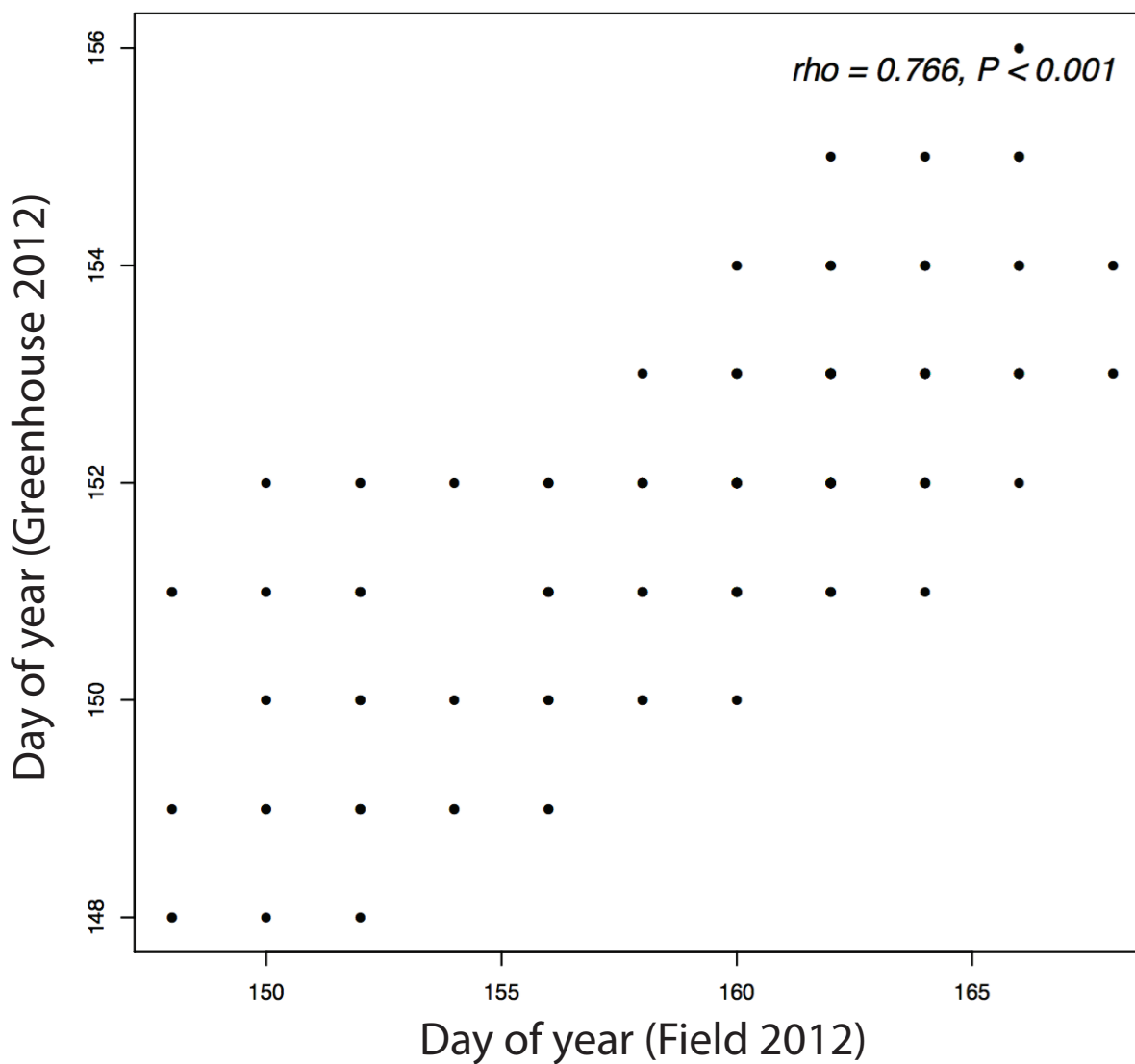

Supplement: S23 Fig — (A) Box plot distributions of the Julian day of sampling for the SwAsp sub-populations. (B) The relationship between Julian day of bud flush for the greenhouse sampled buds and Julian day of bud flush in the field for the same year (2012). (PDF) [file pgen.1006402.s026.pdf]

# SNP Potra004332:6675; Gene Potra003866g23234

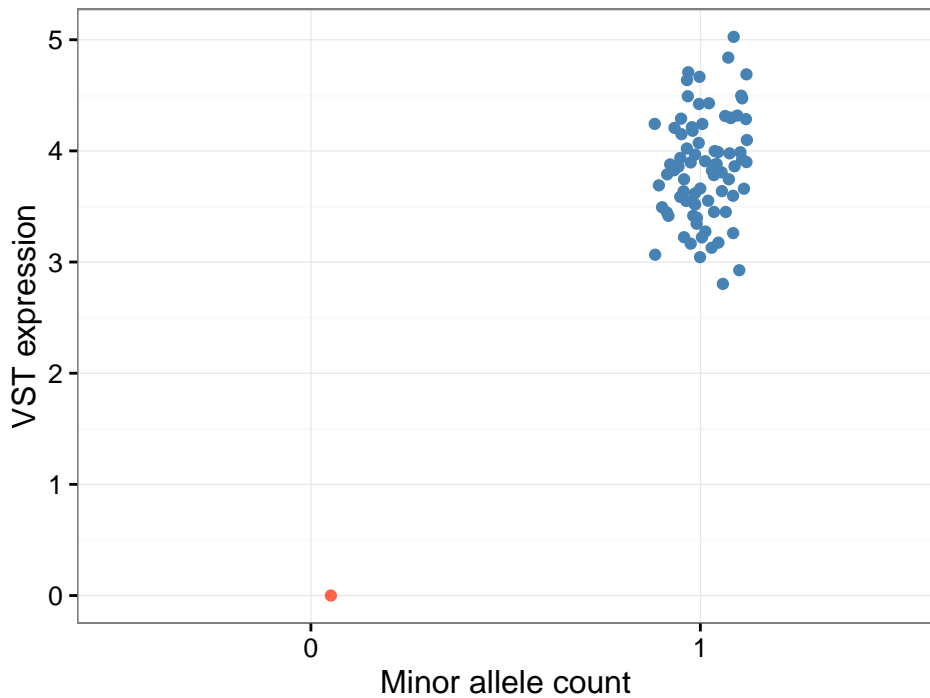

Supplement: S24 Fig — In this case the SNP is heterozygous in all samples but one, resulting in a minor allele frequency close to 0.5, but a major genotype frequency close to one. The resulting association turns out very significant, but is only supported by a single sample. (PDF) [file pgen.1006402.s027.pdf]

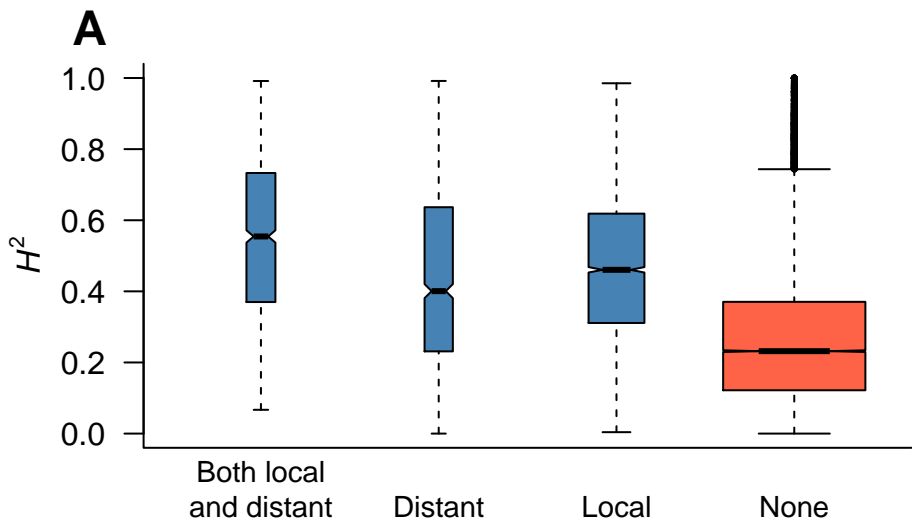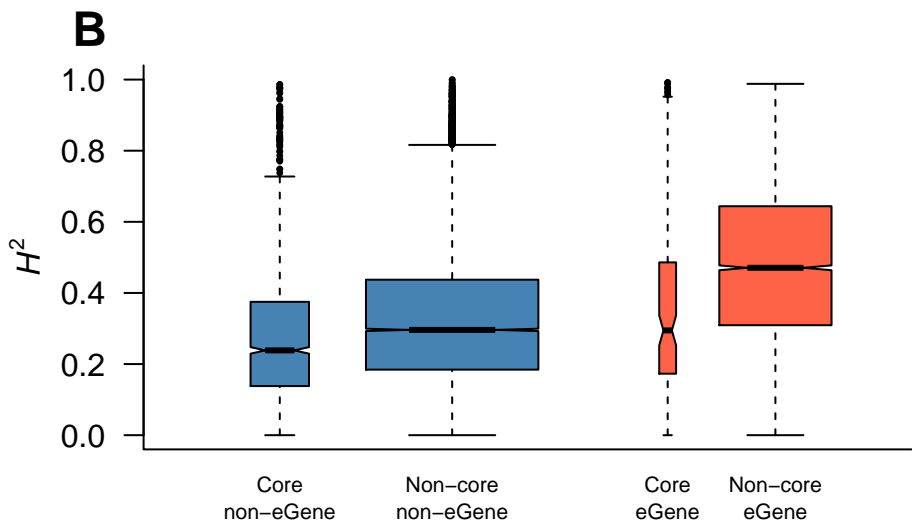

Supplement: S25 Fig — (A) Heritability distributions for different eGene categories: genes with both local and distant eQTLs, genes with only distant eQTLs, genes with only local eQTLs, and genes with no significant eQTLs. (B) Heritability distributions for core and non-core genes divided into whether they are eGenes or not. The box widths are proportional to the number of genes in each set. (PDF) [file pgen.1006402.s028.pdf]
